# Immunologic aspects of migraine: A review of literature

**DOI:** 10.3389/fneur.2022.944791

**Published:** 2022-09-28

**Authors:** Mehrnaz Salahi, Sina Parsa, Delaram Nourmohammadi, Zahra Razmkhah, Omid Salimi, Mohammadreza Rahmani, Saeid Zivary, Monireh Askarzadeh, Mohammad Amin Tapak, Ali Vaezi, Hamidreza Sadeghsalehi, Shirin Yaghoobpoor, Mehran Mottahedi, Setareh Garousi, Niloofar Deravi

**Affiliations:** ^1^Student Research Committee, School of Pharmacy and Pharmaceutical Science, Isfahan University of Medical Sciences, Isfahan, Iran; ^2^Student Research Committee, Kurdistan University of Medical Sciences, Sanandaj, Iran; ^3^Student Research Committee, School of Medicine, Shahid Beheshti University of Medical Sciences, Tehran, Iran; ^4^Student Research Committee, School of Pharmacy, Shiraz University of Medical Sciences, Shiraz, Iran; ^5^Student Research Committee, Faculty of Medicine, Islamic Azad University of Najafabad, Isfahan, Iran; ^6^Student Research Committee, Zanjan University of Medical Sciences, Zanjan, Iran; ^7^Student Research Committee, Kashan University of Medical Sciences, Kashan, Iran; ^8^Department of Immunology, School of Medicine, Shahid Beheshti University of Medical Sciences, Tehran, Iran; ^9^School of Medicine, Tehran University of Medical Sciences, Tehran, Iran; ^10^Department of Neuroscience, Faculty of Advanced Technologies in Medicine, Iran University of Medical Sciences, Tehran, Iran; ^11^Faculty of Medicine, Mashhad University of Medical Sciences, Mashhad, Iran

**Keywords:** migraine, immunology, immunological: autoimmune disease, headache, immunologic aspects

## Abstract

Migraine headaches are highly prevalent, affecting 15% of the population. However, despite many studies to determine this disease's mechanism and efficient management, its pathophysiology has not been fully elucidated. There are suggested hypotheses about the possible mediating role of mast cells, immunoglobulin E, histamine, and cytokines in this disease. A higher incidence of this disease in allergic and asthma patients, reported by several studies, indicates the possible role of brain mast cells located around the brain vessels in this disease. The mast cells are more specifically within the dura and can affect the trigeminal nerve and cervical or sphenopalatine ganglion, triggering the secretion of substances that cause migraine. Neuropeptides such as calcitonin gene-related peptide (CGRP), neurokinin-A, neurotensin (NT), pituitary adenylate-cyclase-activating peptide (PACAP), and substance P (SP) trigger mast cells, and in response, they secrete pro-inflammatory and vasodilatory molecules such as interleukin-6 (IL-6) and vascular endothelial growth factor (VEGF) as a selective result of corticotropin-releasing hormone (CRH) secretion. This stress hormone contributes to migraine or intensifies it. Blocking these pathways using immunologic agents such as CGRP antibody, anti-CGRP receptor antibody, and interleukin-1 beta (IL-1β)/interleukin 1 receptor type 1 (IL-1R1) axis-related agents may be promising as potential prophylactic migraine treatments. This review is going to summarize the immunological aspects of migraine.

## Introduction

Migraine is the sixth most common disease globally, affecting about 15% of the population, and is a leading cause of disability (1). Migraine attacks are known to cause vasomotor lability originated genetically and contribute to neurological, metabolic, and immunological factors. The mystery of how migraine affects our body is not ultimately revealed yet.

The sensory sensitivity that is a typical sign of migraine is probably due to an impairment of the monoaminergic sensory control system, located in the brainstem and the hypothalamus. Experimental studies suggested that neuroinflammatory mechanisms are involved in the pathophysiology of migraine. Preclinical models demonstrated the role of neuroinflammation in activating the trigeminal pathway at numerous peripheral and central sites such as dural vessels, Gasser's ganglion, and the trigeminal nucleus caudalis.

Many studies showed that inflammation, particularly neurogenic inflammation, has been implicated in the pathophysiology of migraine (2). Non-steroidal anti-inflammatory drugs (NSAIDs) have long been used in treating migraine attacks (3), so that multiple cytokines, such as IL-1β, tumor necrosis factor (TNF), and IL-6, have been associated with the pathogenesis of migraine, as their levels are altered in migraine patients (4). Moreover, the evidence of elevation in pro-inflammatory cytokines, the prevalence of T helper 1 (Th1) lymphocytes, and depletion in regulatory lymphocyte subsets in peripheral blood of migraine patients seems to support the role of inflammation in the pathophysiology of this disease. The effect of inflammatory conditions on the pathophysiology of migraine has encouraged the researchers to investigate the human leukocyte antigen (HLA) phenotypes and cytokine polymorphisms to study their possible association with the risk and severity of migraine.

Migraine headaches have also been claimed to be a neurological appearance of many autoimmune and immunological disorders which involve the central nervous system (CNS), such as multiple sclerosis (MS), or diseases with systematic aspects such as systemic lupus erythematosus (SLE) (2, 5–11).

This study aims to discuss the potential immunological aspects of migraine to attain a more comprehensive understanding of the pathophysiology of this disease.

## Materials and methods

This review covers various articles published in English from 1999 to the present, gathered from medical research databases including PubMed and Scopus, using mesh-search of the following terms: immunology—allergy—autoimmunity—migraine—cytokines—chemokines—immunoglobulin E (IgE)—mast cells –inflammation—CGRP. We also searched the references of selected articles and used the “cited by” tool in the Scopus database to find the latest studies.

### Pathophysiology of migraine

Migraine has been a known disease for centuries (12). Over the past 50 years, advances have helped to understand the pathophysiology of migraine (12–14). Scientific findings showed that migraine could be explained by vasodilation and neurological events (13). According to the studies, during migraine attacks, there is no change in the diameter of the external carotid artery system (middle meningeal artery and superficial temporal artery). In contrast, the vessels are significantly dilated during the attacks in the internal carotid artery (intracranial and extracranial internal carotid artery and middle cerebral artery) (15).

On the other hand, the neurovascular hypothesis suggests that the vascular activating neuropeptides, which are released from the end of the trigeminal nerve (trigeminovascular (TGV) system), are involved in the development of migraine (13, 16, 17). In fact, during cortical spreading depression (CSD), activating trigeminal sensory afferents causes the onset of migraine headaches (18, 19). Furthermore, among the three trigeminal nerve branches, the ophthalmic branch contributes most to migraines (20).

Calcitonin gene-related peptide induces the dilation of the vessels, neurogenic inflammation, and synthesis processes in environmental and central events during a migraine attack (14, 16, 20–23). The CGRP molecule, expressed in half of the trigeminal ganglion neurons, is stored in the nerve terminals, and when neurons stimulate, CGRP is released to the synaptic space (24). Studies have shown that during migraine attacks, the levels of CGRP increase in extra-cerebral circulation, external jugular vein, saliva, and cerebrospinal fluid (CSF) (25–28). Also, it is demonstrated that CGRP infusion in migraine patients causes migraine-like attacks (29, 30). As a result, the CGRP is a potential biological marker in acute migraines (31).

Imaging studies have reported an increase in blood flow to the hypothalamus area early stages, so it illustrates the role of the hypothalamus in migraine attacks (32).

### Neuroinflammation and migraine

Many cytokines, including IL-1β, IL-6, and TNF, are associated with the pathophysiology of migraine because their levels vary among migraine patients (33).

However, the involvement of CNS inflammation in the onset of the migraine attacks is not proven, as changes in blood–brain barrier (BBB), leukocyte infiltration, and glial cell activation have not been detected in migraineurs (2). According to the increase in the TGV system-related inflammatory markers, the peripheral nervous system (PNS), particularly the trigeminal ganglion, is suggested to be the site of inflammation (2).

#### Chronification and sensitization

An increase in CGRP levels in plasma from the jugular vein is detected during an attack of migraine (34). CGRP is released directly from the somata of neurons to synaptic sites in the environment (35). Based on the isolated trigeminal neuron recordings, CGRP was excitatory, leading to spontaneous firing or decreasing the threshold for action potentials (36).

According to the *in vivo* studies on Sprague-Dawley rats as a model of trigeminal nerve activation, CGRP might induce continuous activation of satellite glial cells (SGCs) (37) and Aδ fibers in migraine (38, 39). These cells express CGRP receptors in the trigeminal ganglia (40), and such activation is powerfully demonstrated. For instance, CGRP increases dorsal root ganglion (DRG) neuronal soma excitability (36), intraganglionic CGRP injection leads to hyperalgesia (41), and the effect of CGRP stimulation on trigeminal neurons alters the activity of the pain-related molecules of intracellular signaling such as cAMP-response element-binding protein (CREB), cyclic adenosine monophosphate (cAMP), extracellular signal-regulated kinase (ERK), and mitogen-activated protein kinase (MAPK) p38 (42). This activation increases inflammatory cytokine expression in the dura mater and probably in the cell bodies of neurons and the SGCs in the trigeminal ganglion. “Neurogenic neuroinflammation” is the term that defines inflammatory reactions in the nervous system as a response to neuronal activity (2). Chemokines and cytokines are released by neurons, astrocytes, microglia, T cells, and macrophages. These factors might directly or by activating non-neuronal cells trigger nociceptive neurons, depending on which receptors they express (2).

It is postulated that continuous stimulation of C fibers during recurrent migraine attacks, and subsequent activation of SGCs and Aδ fibers, causes neurogenic neuroinflammation in the trigeminovascular system, enhancing the process of chronification (2). The findings suggest that activation of an inflammatory signal pathway dependent on MAPK is involved in CGRP overexpression in nociceptive neurons, which can contribute to pain hypersensitivity (43). It is observed that local inflammation in the temporomandibular joint (TMJ), induced by Complete Freund's adjuvant, leads to an inflammatory response in the trigeminal ganglion, at which the TMJ sensory fibers' cell bodies are located. This mechanism signifies a single anatomical and functional unit involving neurons and SGCs (44).

Given the lack of standard markers of CNS inflammation, such as changes in glial cell activation, BBB integrity, or leukocyte infiltration, involvement of CNS inflammation in the onset of a migraine attack is not suggested. The findings of inflammatory markers in migraineurs could be explained by the idea of neurogenic neuroinflammation, which occurs in the trigeminal ganglion. Neurogenic neuroinflammation due to the continuous release of neurotransmitters can play a crucial role in realizing migraine chronification (2).

#### Cytokines and inflammatory markers

Among sick children, patients without aura had higher amounts of IL-1β than those with aura (45). Still, some studies did not illustrate a significant difference in IL-1β and tumor necrosis factor alpha (TNF-α) amounts between attack-free periods and attack periods (46, 47). Anti-inflammatory cytokines, including interleukin 4 (IL-4) and interleukin 5 (IL-5), decreased within the attack periods (48, 49). Interleukin 10 (IL-10) within attack periods compared with the interictal periods was increased (8, 48, 50). IL-10 decreased by sumatriptan (one of the triptans) (8). The amounts of TNF and other cytokines have been shown during migraine attacks; however, the relevance of these changes to migraine pathophysiology is unclear (51).

IL-6, TNF-α, soluble intercellular adhesion molecule-1 (sICAM-1), and nuclear factor (NF)-κB significantly increased in parallel during 2 h of attack onset in contrast to the time of catheter putting (52). Significant increased CGRP levels after 1 h and the highest level of interleukin 8 (IL-8) at 4 h were seen, although monocyte chemoattractant protein-1 (MCP-1) and RANTES (CCL5) did not significantly alter at any time point (53). The IL-1β levels increased slightly from 1 to 4 h. However, decreased levels of IL-1β at the end of the attack reached similar values at the time of catheter putting (48).

The serum levels of TNF-α in patients with chronic migraine (CM) were normal. However, levels of TNF-α, MCP-1, interleukin 1 receptor antagonist (IL-1RA), and transforming growth factor-β were higher in the CSF of individuals with episodic tension-type headaches and migraine with and without aura compared to those without pain (54). However, there was no adequate differentiation between increased amounts in these headache types (55).

#### Blood–brain barrier permeability

There are few reasons to believe that integrity of the blood–brain barrier (BBB) is affected during an actual migraine attack, as primary headaches have not been linked with the opening of BBB in any clinical studies (56, 57). Furthermore, the BBB was intact during spontaneous migraine attacks without aura (58), and dural inflammation, which was induced, did not affect BBB integrity (59).

#### CNS inflammation: The hypothalamus and limbic system

Schulte and May observed increased hypothalamic activation in the prodromal phase compared to the interictal state as the most robust evidence for hypothalamus involvement in migraine (60). No studies have directly related to hypothalamic neuroinflammation to migraine (2). A study showed that positron emission tomography (PET) could detect hypothalamic inflammation, but PET and other imaging studies in migraine patients did not demonstrate visible inflammatory alterations in the hypothalamic area (61). Hypothalamic activation results in the activation of the trigeminocervical complex (TCC) or the trigeminal nucleus caudalis (TNC), indicating a connection between these regions of CNS in early migraine (60). There is no evidence showing that inflammation occurs in TNC itself in migraineurs. However, some evidence suggested the contribution of inflammatory mediators to TNC sensitization (2).

#### Migraine and neuroinflammation-related genes

Migraine is a complex neurovascular disease with a solid genetic background, meaning it is formed by a combination of multiple genetic and environmental factors (62, 63). In total, three definite hemiplegic migraine genes have been found now, which are CACNA1A (FHM1), ATP1A2 (FHM2), and SCN1A (FHM3). Despite recent technological advances in whole genome/exon next-generation sequencing, no other migraine hemiplegic genes have been identified (64).

A study revealed that cortical chemokine (C-C motif) ligand 2, IL-1β, and TNF-α mRNA expression increased at 1, 2, and 4 h, respectively, caused by non-invasively optogenetic induction of multiple CSDs through the intact skull in Thy1-channelrhodopsin-2 transgenic mice. This response decreased in IL-1 receptor knockout mice, which indicated IL-1β to be an upstream mediator (65). A re-analysis of the data by Eising et al. (66), which investigated alterations of gene expression 24 h after CSD in the transgenic knock-in mouse model in which missense mutation of the human FHM1 R192Q was inserted (67), indicated a greater expression of IL-1 receptor antagonist (IL-1RN). Also, IL-6 expression was higher in the FHM1 mutant mice's brains than in wild-type mice. In contrast, interleukin 2 (IL-2), IL-4, IL-10, and interleukin 13 (IL-13) showed no difference in genotypic expression (68). That study speculated a possible homeostatic role of IL-6 and IL-1RN in ongoing immunoinflammatory events. Eising et al. (66) showed that CSD events produce a unique delayed inflammatory effect determined by interferon-mediated inflammatory signaling. An overrepresentation of interferon-related transcription factor-binding sites [interferon consensus sequence-binding protein (ICSBP), the interferon regulatory factor (IRF), and IFN regulatory factor (ISRE)] in the promoter regions of the found genes confirmed it. In addition, CSD led to a noticeable continued upregulation of genes, including Cd53, Ms4a6d, Ccl2, C3ar1, Anxa2, Timp1, and Vim, which are primary drivers of signaling in inflammation (69).

A comparison between genetic data of 59,674 migraineurs and 316,078 controls performed by a genome-wide association analysis (GWAS) showed thirty-eight genomic susceptibility loci in humans. Among the genomic susceptibility loci, five genes were related to the inflammation, namely, MEF2D, JAM3, TSPAN2, NOTCH4, and NLRP1 (70). Further genetic confirmation for the involvement of neuroinflammation in migraineurs is obtained by comparing the genetic data of 4,505 migraines with aura and 4,038 migraines without aura (and corresponding control sets) in a setting of genome-wide association studies (GWASs), which showed both types of migraine to be more similar rather than being different and that among their genetic overlap, there was a significant number of inflammation-related genes, as well as genes associated with the cardiovascular system and connective tissue (71).

#### Meningeal neurogenic inflammation and nociceptor activation

Significant recent advances in molecular pharmacology have clarified the molecular mechanisms behind neurogenic inflammation. Trigeminal neurons by releasing tachykinins and endothelin-3 (ET-3) on tachykinin receptor 1 (Tacr1) and endothelin receptor type B (Ednrb) on endothelial cells activate them and result in increased dural vascular permeability and vasodilation. Cellular contraction brought on by endothelial cell receptor stimulation results in plasma protein extravasation (PPE), neurogenic inflammation's (NI's) most prominent physiological feature, and nitric oxide-induced vasodilatation. The calcitonin gene-related peptide (CGRP), a crucial component of NI, does not affect vascular permeability; however, it causes neurogenic vasodilatation (NV) by directly relaxing vascular smooth muscle, which is endothelium-independent (72).

At the place where the nociceptive fibers are stimulated, pain is usually accompanied by an inflammatory response in different degrees. Much experimental evidence, particularly from rats, shows that the nociceptive trigeminocervical afferents that mediate headache can be triggered by a sterile meningeal inflammatory process (73). Studies on premonitory symptoms (61) and continuous scanning of the migraine cycle (60, 74) support the current idea that migraine is a brain condition in which attacks are triggered in subcortical areas (61). The discovery of neurotransmitters and neuromodulators that may be involved in the pathogenesis of migraine is a critical problem in migraine research (75). However, the existing clinical data show that substance P is not involved in acute migraine episodes (72) because substance P receptor antagonists have no role in this scenario (76–78). The production of cytokines, chemokines, reactive oxygen species (ROS), and secondary messengers such as nitric oxide (NO) and prostaglandins modulates neuroinflammation in the brain and spinal cord (76, 79). The trigeminovascular system (TVS), which CSD activates, is related to migraine aura development. Trigeminal ganglion neurons' antidromic conduction, opposite to the orthodromic conduction, releases neuropeptides from their nerve terminals, including CGRP, which causes vasodilation and plasma extravasation. Sensory nerve fibers contain neuropeptides coming from the trigeminal ganglion (TG) that innervate the dura, generating neurogenic inflammation in the dura. The direct conduction of TG neurons can activate c-fos in the TNC, resulting in a pain sensation that is eventually experienced as a headache (77). Furthermore, evidence from mice and rat studies showed that parenchymal neuroinflammatory signaling between neurons, astrocytes, and microglia, which finally migrates to the meninges (65, 66, 78, 80–85), might be the potential pathway of transferring a non-homeostatic activity in the unconscious brain to pain-sensitive meninges (86, 87).

According to the rodent experiments, a sterile meningeal inflammation triggered by the release of peptides from trigeminocervical C-fibers and the activation of resident inflammatory cells (mast cells, macrophages, and dendritic cells) may contribute to the sustained activation and sensitization of meningeal nociceptors (16, 88–90). The data imply that this inflammatory signaling occurs in the trigeminocervical ganglia and the meninges. This signaling may be involved in the central nociceptive pathways of headache chronification (91, 92). When meningeal nociceptive fibers are activated for a long time, they can release a variety of vasoactive peptides such as CGRP, pituitary adenylate cyclase-activating polypeptide (PACAP), substance P, and neurokinin-A (73, 93). Research on isolated human middle meningeal and cerebral arteries has shown the vasodilator effects of several neuropeptides (94, 95). Experimental studies have shown that systemically given fremanezumab (CGRP antagonist) did not affect post-CSD middle meningeal artery (MMA) dilation and dural plasma protein extravasation (which has a parallel time course to MMA dilation) but inhibited A-fiber mediated nociception, supporting the theory that MMA dilation is not directly related to nociception (39, 96).

In rats, CGRP alone or the inflammatory mediator prostaglandin E2 could not cause extravasation of dural plasma proteins (97). In humans, except for rare case reports, there is minimal evidence of protein plasma extravasation during migraine (98). Furthermore, even in animal models, CGRP alone does not cause mast cell degranulation (99), with only one research reporting positive findings. Also, the receptor components required for a CGRP response are not expressed in human mast cells (100). As a result, the theory that mast cell degranulation plays a role in migraine episodes in humans does not hold up (2). However, CGRP has a significant role in migraine pathophysiology because CGRP levels in the jugular vein plasma increase significantly during migraine episodes (34). In migraineurs, an intravenous CGRP infusion causes migraine-like symptoms (101), and clinically effective anti-migraine medications target either CGRP release (triptans) or CGRP peptide or its receptor (CGRP antagonists) (102). Mast cells also release tryptase, which generates migraine-like pain responses in mice by activating the protease-activated receptors (PAR) on dural afferents (103–105). Much research in rodents (106) has shown a significant role in neurogenic inflammation in migraine pathogenesis.

#### Inflammation response provoked by CSD

Cortical spreading depression is a characteristic feature of migraine with aura, displaying a powerful wave of neuronal depolarization with vascular and glial activation (107, 108). There is actual documentation that shows CSD causes neuroinflammation, although the suppression of inflammation could not necessarily treat migraine. Studies showed that inflammation of the meninges by stimulating mast cells and macrophages and increasing the amounts of pro-inflammatory cytokines, such as IL-1β, IL-6, and TNF-α, caused the development of CSD (82, 84, 109–111). *In vitro* analysis of spreading depression in hippocampal organotypic cultures (112) and astrocytes (84) illustrated the upregulation of pro-inflammatory cytokines, including IL-6, IL-1β, and TNF-α.

**Figure 1:**
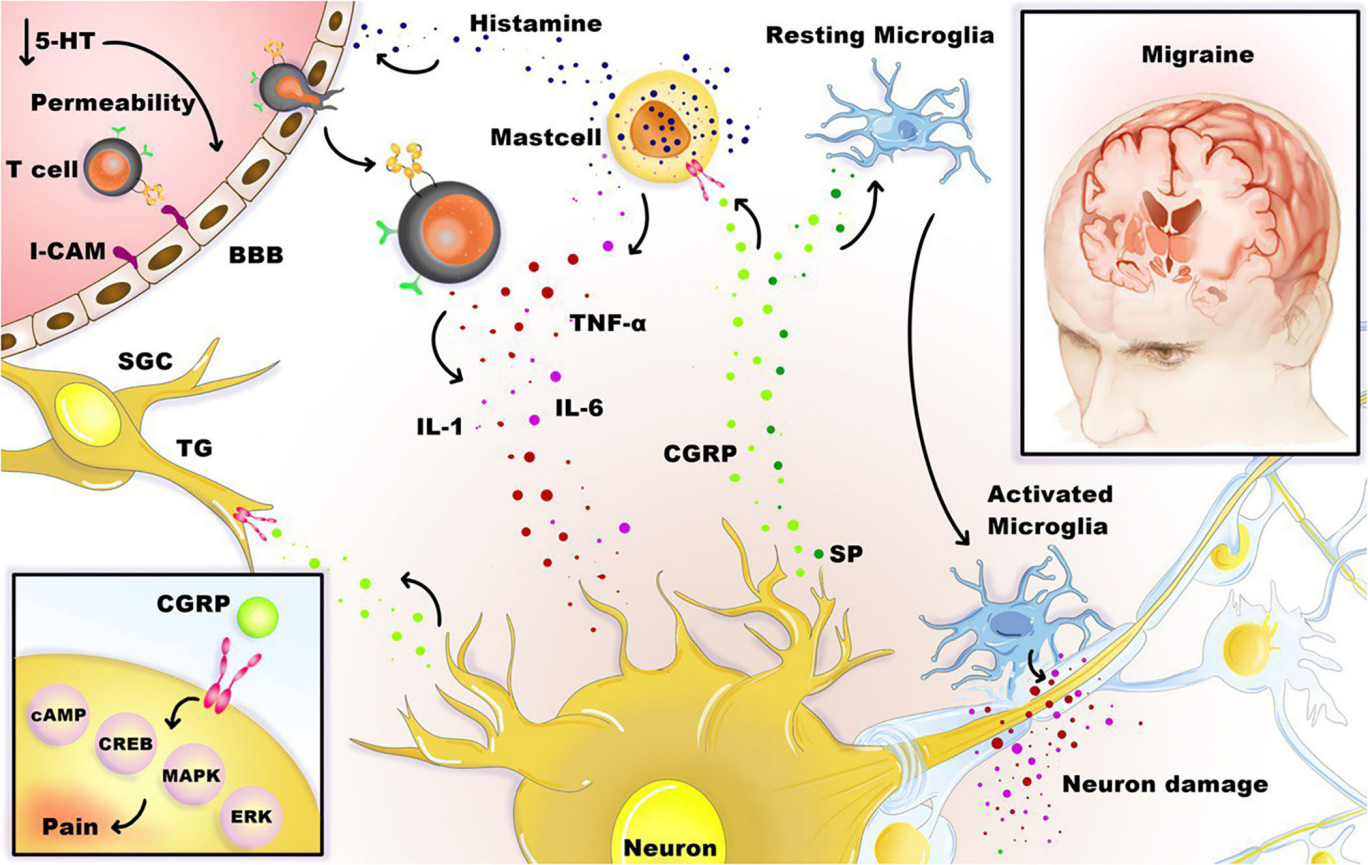
Neuroinflammation and migraine. Stimulation of the trigeminal neurons causes the release of neuropeptides, including CGRP, substance P (SP), leading to mast cell degranulation, leukocyte infiltration, glial cell activation, and increased production of inflammatory TNF-α, IL-1, and IL-6 cytokines. Besides, satellite glial cells (SGCs) and trigeminal ganglions (TG) express receptors for CGRP, and CGRP can stimulate intracellular signaling molecules that are relevant to pain, such as cAMP, CREB, MAPK, and ERK. Under the influence of inflammation, activated microglia, T cells, and mast cells can boost the inflammation loop and production of cytotoxic mediators in the CNS.

Figure 1 illustrates the association between neuroinflammation and migraine.

### Inflammasomes in migraine

#### General concepts of inflammasomes

Pattern recognition receptors (PRRs) detect pathogenic molecular patterns (PAMPs), and the environment- or host-derived danger-associated molecular patterns (DAMPs) detect pathogenic signals. In the CNS, the PRRs are mainly found in microglia, astrocytes, and macrophages, and also, neurons, endothelial cells, and oligodendrocytes have PRRs (113, 114). PRRs are found at the cell membrane and in the cytoplasm. PRRs at the cell membrane are Toll-like receptors (TLRs), which detect extracellular signals. PRRs in the cytoplasm include nucleotide-binding domain and leucine-rich repeat-containing receptors (NLRs) and A melanoma 2 (AIM2)-like receptors (ALRs), and they detect intracellular signals. “Inflammasomes” that are part of the innate immune response are created by intracellular PRRs (115).

So far, few rodent studies have sought the association between inflammasome and migraine. One study showed that NLR family pyrin domain containing 3 (NLRP3) inflammasome pathway involved in the peripheral trigeminal ganglion (TG) response of the intracranial pain model induced by inflammatory dural stimulation in the rat (116). Another study revealed an increase in the production of NLRP3 inflammasome and IL-1β activation in a migraine-related pain mouse model (induction of pain by repeated nitroglycerin (NTG) stimulation) (117). It also showed that blockade of NLRP3 and IL-1β reduced hyperalgesia and prevented increased markers related to chronic migraine central sensitization such as c-Fos, CGRP, and phospho-ERK (p-ERK) in the trigeminal nucleus caudalis (115). A review suggested the involvement of D-β-hydroxybutyrate (D-BHB), as a ketone body, in the pathophysiology of migraine, that is, mitochondrial function, oxidative stress, inflammation, cerebral excitability, and the microbiome of the gut (118). No data confirm the relationship between cortical spreading depolarization (CSD) or migraine and NLR family pyrin domain containing 1(NLRP1) or NLR family pyrin domain containing 2(NLRP2) inflammasomes. Therefore, the role of NLRP1, NLRP2, NLRP3, AIM2, and other inflammasomes in migraine with aura (MA)-associated parenchymal neuroinflammation, for instance, in response to CSD, needs to be investigated in the future studies (115).

#### Inflammasome complex formation and release of IL-1β and HMGB1 from neurons

More studies illustrated that some inflammatory mediators, including IL-6, IL-1β, TNF-α, prostaglandin E2, and nitrite levels in the internal jugular vein (which drains the brain parenchyma but not the meninges) increased within the first hour of a moyamoya (MO) attack (52, 53, 119). High expressed DNA-binding non-histone proteins called high mobility group box protein 1(HMGB1) (120) are responsible for chromosome stabilization, DNA repair, control of transcription by binding to DNA, and nucleosome mobility (120). These alarmin proteins are passively released from necrotic or damaged cells and actively leak after an inflammatory condition such as infection, cell swelling, and tissue injury (121, 122) and provoke a rapid inflammatory response following release from the cell (123). The release of HMGB1 and IL-1β stimulates the nuclear factor-κB (NF-κB) pathway in adjacent cells, and this pathway regulates the neuroinflammatory response in astrocytes and microglia (124, 125).

#### Mitochondria, migraine, and the inflammasome

Abnormalities of mitochondria have been identified in patients with migraine, as indicated by directly observing biopsies of muscle that showed giant mitochondria with paracrystalline inclusions, cytochrome-c oxidase-negative fibers, and ragged red, and the gathering of subsarcolemmal mitochondria (126, 127). Common polymorphisms of mitochondrial DNA (mtDNA) (3010G-A and 16519C-T) are associated with migraine and pediatric cyclic vomiting syndrome (128). Moreover, various missense mutations of POLG have been linked to migraine (129). However, the importance of these variants for migraine pathophysiology is unclear. Perhaps unsurprisingly, a current migraine mitochondrial GWAS failed to find a genetic factor (130). A high migraine headache frequency was present in Mitochondrial Encephalopathy, Lactic Acidosis, and Stroke-like episodes (MELAS) patients with mutation m.3243A> G (131). This mutation in endothelium and vascular smooth muscle cells, glial cells, and neurons causes long-term subjection to toxic materials, such as reactive oxygen species (ROS), which might contribute to migraines at older ages. Besides high ROS production, narrowing the vascular lumen and the subsequent peroxidation of lipid, ionic homeostasis, hypoxia/ischemia, and altered glutamate metabolism may be associated with CSD stimuli. These pathways, including the inflammatory pathways caused by the production of ROS, have been proposed as new targets for new drug classes for the treatment of mitochondrial migraine in patients with m.3243A> G (131). Mitochondria also interact with the immune system, i.e., with inflammasome induction. Mitochondrial ROS (mtROS) that damages mtDNA and interacts with NLRP3 inflammation through inflammatory events can be produced by mitochondria (132, 133). The overproduction of mtROS is detected by mtDNA or thioredoxin-interacting protein (TXNIP), which attaches to the leucine-rich replication repeat of NLRP3, thereby activating the NLRP3 inflammasome (133). Activation of inflammasome and ROS production was reduced by rotenone, an inhibitor of mitochondrial I complex (134). Mito-TEMPO, a specific mitochondria ROS scavenger, stopped the release of mtROS, consequently reducing the positive regulation of interleukin 18 (IL-18) and IL-1β induced by lipopolysaccharide/ATP or ethanol and restraining the activation of NLRP3 inflammasome (135).

#### Cholinergic anti-inflammatory pathway and the inflammasome

Extracellular ATP induces a rapid influx of acetylcholine (Ach) into the cytoplasm. ACh stops the release of mitochondrial DNA and NLRP3 ligand *via* Alpha7 nicotinic acetylcholine receptor (α7 nAChR), thus suppressing the activation of NLRP3 inflammasome in peritoneal macrophages of lipopolysaccharides (LPS)-primed mice (136). In these settings, vagus nerve stimulation (VNS) or cholinergic receptor agonists significantly inhibit inflammasome activation, whereas inflammasome activation is notably increased by genetic ablation of α7 nAChR.

### Pharmacotherapy in migraine

Migraine management began with ergot alkaloids, followed by the emergence of the triptans, and later expanded to other targets of therapeutic type, particularly calcitonin gene-related peptide (CGRP)-related products (137, 138). Significant recent advances in molecular pharmacology have clarified the molecular mechanisms behind neurogenic inflammation.

Triptans mainly target serotonin 1A receptor (5-HT1) receptors as an agonist (139, 140). 5-hydroxytryptamine (serotonin) receptor 1D (5-HT1D) receptors inhibit neuropeptides' release in guinea pigs, which leads to the modification of the dural response of the neurogenic inflammatory type (141). Serotonin-1F receptor (5-HT1F) receptors can be selectively activated by Ditans (142), inhibiting CGRP release and possibly substance P from the peripheral endings of the trigeminal nerve in the dura mater and affecting the thalamus or the caudal trigeminal nucleus (143).

Non-steroidal anti-inflammatory agents (NSAIDs) and ergot alkaloids as treatments for headaches in migraine may reduce the neurogenic inflammatory response (144). NSAIDs disrupt the synthesis of prostaglandins involved in hyperalgesia and the inflammatory cascade by impeding the function of the enzymes cyclooxygenase-1(COX-1) and cyclooxygenase-2 (COX-2) (145, 146). The endocannabinoid system could modulate the neurogenic-induced migraine (147) based on cannabinoid receptor type 1 (CB1) receptors localization along the trigeminal tract and its afferents (148, 149).

Calcitonin gene-related peptide secretion in pathogenesis of migraine includes the regulation of sensory processing (150) and peripheral vasodilation, which leads to the mediation of meningeal neurogenic inflammation *via* release of other neuropeptides (151, 152). CGRP-targeted monoclonal antibodies (mAbs) and gepants, as small-molecule antagonists, are two groups of therapeutic agents that have been elaborated to date to disrupt the function of CGRP. Currently, three gepants and four CGRP-targeted mAbs are approved by the United States (US) Food and Drug Administration (FDA) for treating migraine (153); three of the CGRP-targeted mAbs are against CGRP, and the other one is against the receptor of CGRP (154).

In addition to CGRP, the neuropeptide PACAP has also received attention as a possible anti-migraine target (155). Early research on neuropeptides and migraine showed that only CGRP was raised during a migraine attack (34). However, a further investigation found higher PACAP levels during the ictal period compared to the attack-free time (156, 157), and patients with migraines experienced migraine-like symptoms after injections of both CGRP and PACAP (158). PACAP may appear before CGRP and be significant during the initial stages of a migraine episode. After administering vasoactive intestinal peptide (VIP) and PACAP to migraine sufferers, VIP did not cause a migraine attack, in contrast to PACAP (159, 160). PACAP and VIP have a similar ability to engage VPAC1 (VIP and PACAP receptor 1) and VPAC2 receptors. However, PACAP has a significantly stronger affinity for binding to VPAC1 than VIP (161). These findings suggest that the VPAC1 receptor is the most plausible pathway by which PACAP induces migraine. As a side point, it appears that only VPAC1/2 receptors in investigations of dural vessels are causing dilatation (162), indicating that the PACAP migraine mechanisms are probably unrelated to vasodilation. Amgen developed and began clinical testing a mAb against the VPAC1 receptors (AMG301) (38). A mAb directed against PACAP-38 has been developed and is undergoing clinical testing by Leder, similar to the CGRPergic system (ALD1910). Recent data from preclinical investigations on ALD1910 in a umbellulone-induced rat model of neurogenic vasodilation and parasympathetic lacrimation demonstrated promising outcomes (163).

Ghorbani et al. (164) aimed to assess the effects of supplementation with vitamin D3 on characteristics of headache and pro-/anti-inflammatory markers in patients with migraine, considering the anti-inflammatory effects of this vitamin. In total, eighty episodic patients with migraine were studied double-blindly, randomly divided into two groups one group daily received vitamin D3, and a placebo was provided for the other group. Determination of headache characteristics using diaries and assessing levels of IL-6, IL-10, cyclooxygenase-2 (Cox-2), and inducible nitric oxide synthase (iNOS) in serum were performed at baseline and the end of the study. The results showed that the group that received vitamin D3 supplementation experienced a reduced duration of attacks, fewer headache days per month, headaches with lower severity, and lower painkiller consumption per month compared to the placebo group. Vitamin D3 could be thought of as a regulator of the proliferation, differentiation, and activation of inflammatory and immune cells such as macrophages considering expressed nuclear receptor of vitamin D3 in these cells, and stimulated macrophages might synthesize the main metabolite of vitamin D3 as known as 1,25 (OH)2 D (165, 166). Anti-inflammatory effects of vitamin D3 could be applied by suppressing the activity of NF-κB through various mechanisms, such as stimulating its inhibitory protein (IκB) production (165, 166). NF-κB is a critical transcription factor in regulating inflammatory cytokine synthesis and secretion, including nitric oxide (NO), iNOS, and IL-6. According to these findings, increased activity of NF-κB and levels of NO and iNOS in ictal phases in migraineurs can be alleviated by vitamin D3 supplementation and lead to reduced inflammation in migraine (52).

Zareie et al. (167) assessed the effect of cinnamon on the inflammatory status and migraine attacks. They randomly divided fifty migraineurs into two groups; the experimental group received daily cinnamon powder, and the control group received a placebo for 2 months. They measured the serum levels of CGRP, IL-6, and NO at baseline and the end of the trial. A questionnaire was also used to record the frequency of pain attacks, duration, and severity. Significant reduction in serum IL-6 and NO concentrations and frequency, duration, and severity of migraine attacks were observed in the group that received cinnamon compared to the control group. However, serum CGRP levels had no change in any of the groups. Cinnamon has neuroprotective and anti-inflammatory roles (168, 169). Cinnamaldehyde, as the main bioactive component of cinnamon, can decrease the inflammatory cytokines, including TNF-α, IL-6, and IL-1β, by inhibiting the expression of nitric oxide synthase (iNOS) and cyclooxygenase (168, 170–173).

Moreover, a possible regulating role for cinnamon in inflammatory mediator's release is revealed in the animal studies (174, 175). They also demonstrated the beneficial effects of cinnamon on migraine complications by influencing IL-6 (167) and NO. NO has a role in pain processing (176, 177). Considering the pro-inflammatory effect of NO in inflammatory pain, inhibition of NO production improved neuropathic and inflammatory pain (178). Therefore, NO levels reduction may be considered a pain and other migraine complications reliever (167). Cinnamon reduces NO metabolites, including peroxynitrite and superoxide, reducing NO-induced inflammation and pain (168, 179, 180).

### Migraine and immunologic disorders

**Figure 2:**
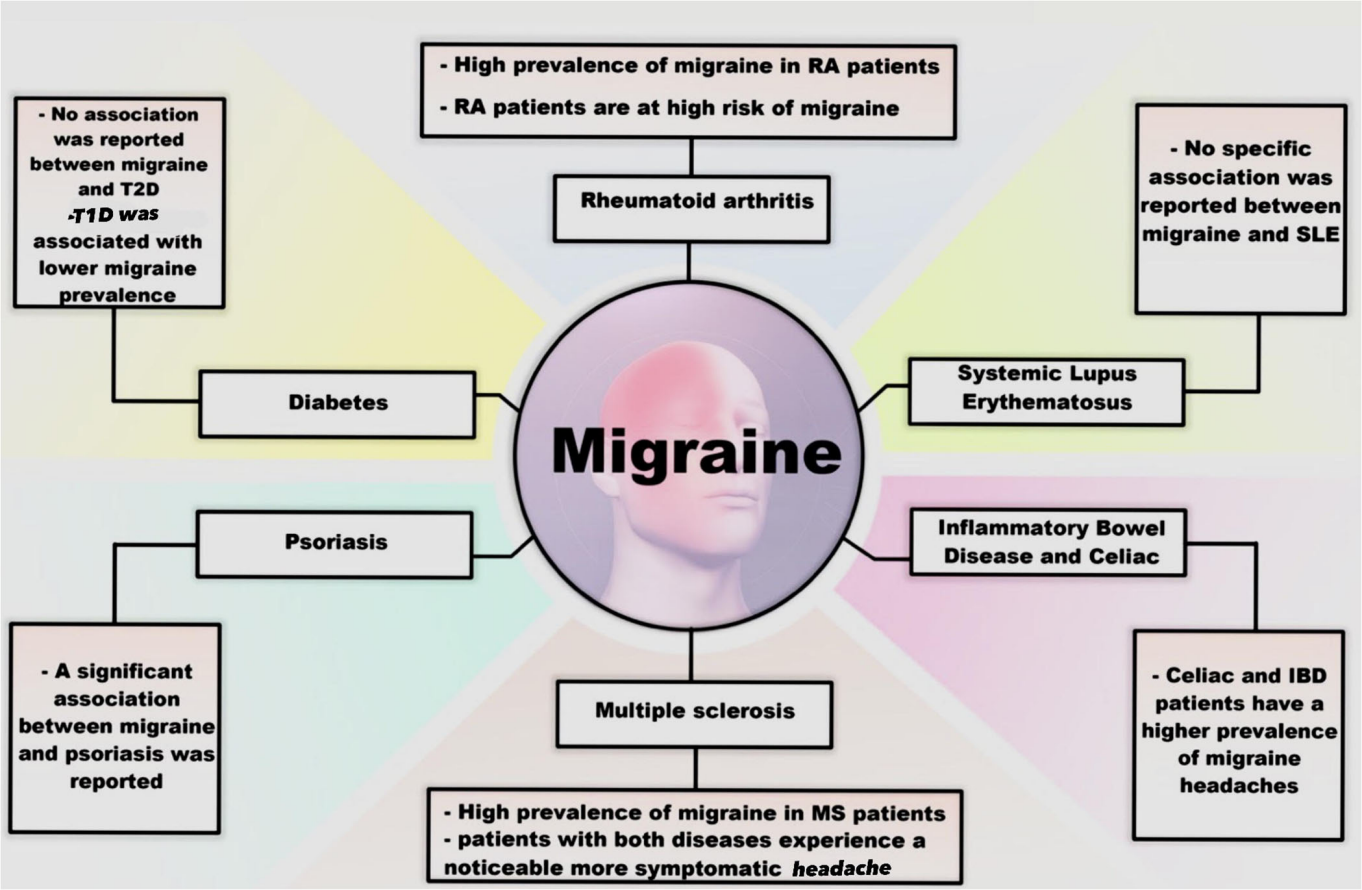
The association between migraine and autoimmune disorders.

Several studies have investigated the links between migraine and immunologic disorders, including multiple sclerosis (MS), rheumatoid arthritis (RA), systemic lupus erythematosus (SLE), type 1 diabetes mellitus (T1D), and psoriasis (Figure 2).

### Migraine and multiple sclerosis (MS)

Overall, 29–86% of patients with MS suffer from pain (181, 182). Although pain is not a key symptom for MS, many patients complain of various pains. One of the pain syndromes linked to MS, which make up between 11 and 37% of the symptoms of MS, is headache (183, 184). Recent research has found that the prevalence of headaches in patients with MS varies greatly (185). Depending on where the lesions are located, patients with MS can show various neurological symptoms, including visual problems (optic neuritis), sensory or motor difficulties, and cognitive dysfunction.

#### Migraine and MS: Prevalence

Several studies have investigated the prevalence or incidence of migraine headaches among patients with MS. Fragoso et al. (186) showed that 746 patients (625 women and 121 men) had both MS and headaches. Among them, 54.1% (404 patients) were diagnosed with migraines, and 68.3% had a moderate to severe disease burden observed in 68.3% of those patients. Also, a 3-fold higher prevalence of migraine among patients with MS compared to controls was reported by a cross-sectional study by Kister et al. (187). Also, a study by Watkins et al. (188) confirmed a higher incidence of migraine among patients with MS than in controls, and a higher incidence of migraine was observed in women compared to men. Family history of migraine showed a higher incidence in patients with MS than in controls. Few cases of the MS patients' group already had MS symptoms after experiencing migraine for the first time. These findings suggest that the disease stress due to the neurological disability is probably involved in migraine development. Conversely, several patients with MS had migraines before MS onset; therefore, these cases do not support the idea explained before. It is shown that some treatments for MS, such as interferon-beta (189, 190), fingolimod, and natalizumab, might increase the frequency and severity of migraine attacks in patients with MS, and migraine certainly is a frequent type of primary headache reported in patients suffering from MS (186).

Moisset et al. designed a cohort study that measured the comorbidity of migraine and pain with neuropathic characteristics (NC) in MS using a questionnaire. Out of 1,300 patients, 79% had experienced pain in the past month, 51% had pain with neuropathic characteristics (NCs), 46% had migraines, and 32% had both migraines and neuropathic characteristics (NCs) at the same time. Patients with migraines compared to rest were young, their disease duration was short, and most had relapsing remitting MS (RRMS) and a lower Expanded Disability Status Scale (EDSS). Higher EDSS and MS duration were associated with a reduced rate of migraine. The prevalence of migraine was age-related and decreased with aging, whereas neuropathic characteristics (NCs) were not related to age (191). The inflammatory phase of MS was associated with migraines (192), and it seems that CNS lesions can also cause neuropathic pain (191).

However, Gustavsen et al. reported no significant difference in the prevalence of headaches and migraines between patients with MS and controls. Patients with EDSS≥4.0 had few migraines. It might be due to the older age of participants compared to the participants in other studies (193). Also, Katsiari et al. showed no link among MS disease activity, clinical manifestations, and autoantibody and the presence or type of headache in patients with MS. This study concluded that migraine should not be considered a neurologic indication in MS (194).

#### Migraine and MS: Structural changes

Several studies have investigated the structural changes explaining migraine in patients with MS. Pravatà et al. designed a study to determine whether changes in the resting-state functional connectivity (RS-FC) will distinguish the occurrence of migraine in patients suffering from MS. They reported that the loss of periaqueductal gray matter (PAG) negative connectivity with the sensorimotor and visual network was related to the severity of migraine symptoms and their impact on daily activities of patients with MS (195).

Tortorella et al. (196) assessed the presence and frequency of structural abnormalities detected by magnetic resonance imaging (MRI) in patients with migraine and MS. They recorded the presence of hyperintense lesions implicating the brainstem structures by brain dual-echo scans taken from a group of patients with migraine (with or without aura), a group of MS patients with migraine not experiencing aura, and a group of MS patients without migraine. Substantia nigra (SN), red nucleus (RN), and PAG lesions were observed in all the groups of patients, with some differences in these regions. Aura presence in migraine patients made no difference compared to those without aura. The RN and SN were more involved in MS patients with migraine than those without migraine. The SN and PAG were noticeably involved in MS patients with migraine compared to migraine patients. Results of the brainstem structures lesions assessment illustrated that migraine aura is not correlated with increased involvement of these structures. An explanation for these observed lesions is that they might be due to repeated blood flow reductions resulting in ischemia (197, 198). Based on the lesions of brainstem nuclei observed in migraineurs, damage to these structures is suggested to play a role in migraine genesis with the independence of the causative lesion.

Children have a higher incidence of headaches at the first clinical event of MS than adults, and the frequent involvement of the brainstem in pediatric MS and the existence of large white matter lesions could explain it. Mariotti et al. reported a 5-year-old case of MS who presented a migraine-like headache at both the initiation of the disease and the subsequent two relapses. In this case, the neuroradiologic findings did not fade with time l/ with each relapse, which explains the persistence of headache as the primary complaint during her two subsequent MS exacerbations. The anatomical location of the lesions may influence the presence of a headache with MS. Most of the essential structures for migraine were implicated in the inflammatory process in the brain and cervical spinal cord in the MRI results. MRI revealed minimal effacement of subarachnoid spaces and some modest evidence of leptomeningeal engorgement during the first attack and the first relapse, indicating inflammatory changes in the meninges. The initial and subsequent MRIs revealed diffuse brain edema. The relief of pain following lumbar puncture revealed a role of brain swelling as a pathogenic cause of headache in the patient (199).

#### Migraine and MS links: Potential mechanisms

Probable mechanisms might be responsible for these results. Migraine can increase the permeability of the BBB and neuroinflammation (200), thereby exposing antigens derived from the sequestered CNS compartment to circulating T cells and sensitizing them to myelin products. Migraine also may change cytokine profile in a manner that causes autoimmune reactions in CNS; for example, IL-10 and TNF-α tend to be enhanced in both migraine attacks and during an MS relapse (4, 201).

Demetgul et al. (202) focused on studying the prevalence of primary headaches in MS sufferings and discovered the type of headache in these patients and found out the relationship between primary headache type and MS subtype and the correlation between the localization of plaques in the brain magnetic resonance imaging (MRI) with clinical findings in MS. The patients with MS were asked about headache features in succession to characterize headache type. The results showed that the mean EDSS score in patients with tension-type headache (TTH) and patients with migraine-type headache was 4.7 and 1.8, respectively, and was statistically significant. Of the participants with migraine-type headaches, 100% had pericallosal, 98% had juxtacortical, 45.9% had cerebellar, and 78.6% had infratentorial lesions. The mean total number of cerebral lesions in patients with TTH was significantly higher than migraine-type headaches. The mean number of brain lesions in patients with headaches was significantly higher than in those without headaches. Migraine was common in patients with sensory, cranial, optic, or cerebellar symptoms at baseline. At the same time, TTH was common in polysymptomatic patients and patients with motor symptoms at baseline.

Gee et al. examined whether or not the prevalence of migraine-like headaches in MS suffering correlates with a plaque in the brainstem. Approximately 17% of subjects were diagnosed with secondary progressive MS, 17% with primary progressive MS, and 66% with RRMS (203). Along the lines of a former study (52%) (204), 55.6% of people suffered from headaches, and separately 61.7% had features of migraine-like headaches, 25.3% of tension-type headaches, and 13% of migraine and tension-type headaches. Subjects with a plaque within the midbrain/periaqueductal gray matter (PAG) areas compared with MS subjects without had a significantly 4-, 2.5-, and 2.7-fold increases in migraine-like, tension-type, and combination of migraine and tension-type headaches. Patients with MS with three or more lesion locations, contrary to patients with MS with 0 to 2 locations, were established to be nearly two times more to have migraine-like headaches, but it was not statistically significant. There was a significant linear tendency between the number of lesion locations and migraine-like headaches. To summarize, midbrain plaque in MS patients is associated with an increased likelihood of migraine-like headaches (203). Different parts of the midbrain [dorsal raphe nucleus (DRN), locus coeruleus (LC), and periaqueductal gray (PAG)] have roles in migraine pathogenesis (205–209). In patients with MS, demyelinating process affects PAG similar to white matter (210–220), but other brain structures such as substantia nigra, red nucleus, and hypothalamus could be involved in migraine (221–223).

Migraine headache in MS suffering may be a sign of relapse or may even notify the onset of the severity of manifestations of MS (224, 225). Freedman and Gray suggested that demyelinating lesions in the brainstem may trigger a migraine center or interlope with inhibitory modulating impulses to activate the vascular changes that occur at the beginning of migraine headaches (224). On the other hand, they suggested that inflammatory reactions could release vasoactive substances such as ATP, bradykinin, and vasoactive amines during a relapse and provoke a migraine attack. In addition, Sicuteri believed that cerebral 5-hydroxytryptamine (5-HT) deficiency decreases the pain threshold and causes migraine headaches (226, 227). Some observations that support the 5-HT hypothesis are that the symptoms were relieved after receiving the 5-HT precursor tryptophan. During a migraine attack, plasma 5-HT levels (227, 228) and CSF concentrations of 5-hydroxy indole acetic acid (5-HIAA) diminish (229–232), and urinary 5-HT and 5-hydroxy indole acetic acid (5-HIAA)—its major metabolite—may increase (227, 228); in addition, nightly migraine generally occurs during rapid eye movement (REM) sleep when plasma 5-HT levels suddenly fall (233, 234). Pharmacological studies using selective 5-HT receptor agonists and antagonists to provoke or diminish migraine headaches showed more information about the role of 5-HT in migraine (228, 235–241). 5-HT deficiencies may be responsible for increased permeability of the BBB and then increase susceptibility to relapse of MS and occurrence of migraine (228, 242–246); in addition, the pineal gland's melatonin could interact with the 5-HT system and may be responsible for the beginning of relapse of MS along with the occurrence of a migraine headache (247–261). Moreover, experimental animal studies showed the role of 5-HT in the pathogenesis of MS (247, 262–265).

**Figure 3:**
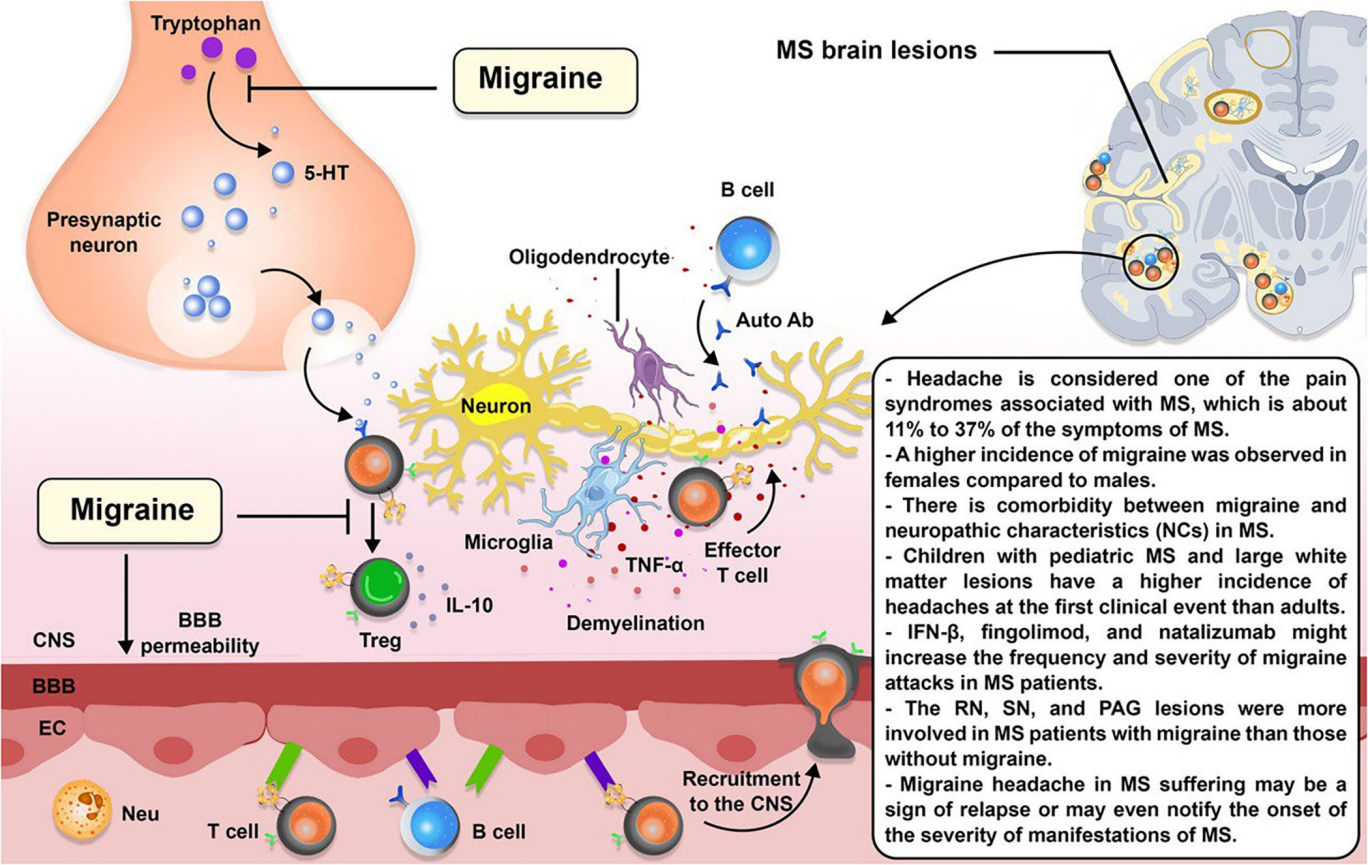
The schematic illustration of the association between MS and migraine. Migraine is a forecaster of MS. 5-hydroxytryptamine (5-HT), a compound produced in the body from the tryptophan, is diminished during migraine headaches. 5-HT induces T cell differentiation to Treg cells; therefore, 5-HT deficiency may affect T cell differentiation toward effector T cells. 5-HT deficiencies may also be responsible for increased permeability of the blood–brain barrier (BBB), resulting in the recruitment of more inflammatory immune cells to the CNS.

Figure 3 indicates a schematic association between migraine and MS.

### Migraine and rheumatoid arthritis (RA)

Studies have shown that RA, a disease of inflammatory synovitis in joints, is more prevalent in patients with migraines (266).

Yoo Hwan Kim et al. (267) reviewed two longitudinal follow-up studies examining migraine's association with RA. Group1 evaluated the risk of RA in people with migraine, and group 2 examined the risk of migraine in people with RA. The outbreak of RA in the migraine group was higher than in the control I group, and the outbreak of migraine in the RA group was higher than in the control II group. In general, migraines increase the risk of RA, and RA is also associated with an increased risk of migraines.

Also, a cross-sectional study by Jacob et al. on 2,649 adults (268–270) showed a significant association between migraine and arthritis (OR = 1.83, 95% CI = 1.20–2.81), especially in women aged <45 years and more than 65 years old. Several points may explain the identified relationship between arthritis and migraine. First, they have expected consequences such as stress, neck pain, and sleep disorders. The second exercise is recommended as a non-pharmacological treatment for both. Overall, the fact that migraine and arthritis may coexist (271) leads to medical and economic burdens and should be planned for (272).

El-Sonbaty et al. realized that the relationship between migraine and rheumatoid arthritis (RA) is complex and not completely understood (273, 274). Therefore, they conducted a cross-sectional study (275) on 210 consecutive patients with RA from Egypt within 6 months. They also observed brain MRI white matter hyperintensities (WMHs) and found out that more disease activity, fibromyalgia (276) and functional losses, longer migraine duration, longer rheumatoid duration, and elevated erythrocyte sedimentation rate (ESR) were considerable in patients with both diseases (275).

Yoo Hwan Kim et al. suggested a bidirectional association between migraine headache and rheumatoid arthritis on the ordinary pathophysiologic mechanisms of the immune system between migraine and RA (271, 277, 278). To test this theory, they designed two longitudinal follow-up studies measuring the risk for RA in persons with migraine and the risk for incident migraine in persons with RA (279, 280). Because the incidence of both diseases was higher in both studies than in the control group, they concluded that migraine and RA exacerbate each other. The pathophysiologic mechanism of inflammation, vascular endothelial cells, and the immune system between mentioned diseases could lead to the two-sided relation between migraine and RA. Wang et al. investigated the prevalence of rheumatoid arthritis in migraineurs in a cohort study. The occurrence rate of RA was expressed at 3.18 per person-years for the migraineurs group and 1.54 per person-years for the non-migraineurs group (271).

Although the precise mechanism that supports the relation between migraine and RA has not been known yet, two conceivable explanations are provided. First, the relation between RA and migraine may be due to a common pathogenic mechanism called dysfunction of the serotonergic system. Second, the high risk of RA in migraineurs may be due to the high prevalence of sleep disorders. As a result, migraine can increase the risk of developing RA in migraineurs (271).

### Systemic lupus erythematosus (SLE) and migraine

Systemic lupus erythematosus is a multi-organ autoimmune disease. The most important clinical features are mucosal, skin, joints, kidney, serous, hematological, immunological, and neurological involvements. CNS involvement can occur in 14–75% of patients with SLE (281, 282). Headache is one of the most common neurological findings in 32 to 78% of patients with SLE (283–292).

In a study by Bicakci et al., no link was discovered between headache characteristics (duration of history, therapeutic agents, location, accompanying signs, course, kind of pain, and family history) and the existence and size of cerebral lesions in patients with SLE. As a result, headache in SLE and its accompanying symptoms did not provide clues about the intracranial lesion. However, if the onset of headache and SLE co-occurs in elderly individuals with long-term SLE, a link could be considered (293).

A meta-analysis in 2004 showed no specific pathogenic mechanisms of headache in adult patients with SLE, and no association was found between headache and disease status, including CNS involvement (294). Also, no association was found in the prevalence of headaches between patients with SLE and controls (294).

In a study by Katsiari et al., the prevalence of chronic tension-type headache (CTTH) in patients with SLE was significantly higher than in controls at baseline and during the 1-year follow-up. There was no difference in the prevalence of migraine (with or without aura) between the MS group (23%), the SLE group (21%), and the control group (22%). The severity of migraine attacks in SLE was lower than control and MS. There was no link among disease activity, clinical manifestations, and autoantibody and the presence or type of headache in patients with SLE or MS. Lower quality of life, anxiety symptoms, and depression were higher in SLE sufferings than in patients with MS or controls but were insignificant (194). Appenzeller and Costallat showed that migraine was prevalent among SLE suffering and was associated with Raynaud's phenomenon, anti-phospholipid antibodies, disease activity, and organ damage. Still, these study's subjects were not wholly homogenous; only premenopausal women participated, and headache diaries were not used (295). However, migraine is a CNS indication of SLE (296), and SLE affects the brain; recent studies did not show a correlation between brain lesions and any headache type in SLE (293, 297–299).

### Migraine and type 1 diabetes mellitus

Diabetes mellitus (DM) is a metabolic disease characterized by hyperglycemia due to defective secretion and insulin function. This blood sugar status is associated with damage to multiple organs and dysfunction, heart, blood vessels, nerves, eyes, and kidneys. DM type 1 (insulin-dependent), DM type 2 (non-insulin-dependent), and other specific types are known as different forms of the disease (300).

Hagen et al. (301) conducted a study to assess the associations between type 1 and 2 DM and migraine. Adjusted analysis of the 26,121-participant group revealed an association of classical type 1 DM with lower headache and migraine prevalence compared to those without experiencing DM. Similar results were obtained in the merged group of autoimmune diabetes of adults [latent autoimmune diabetes in adults (LADA)] and classical type 1 DM. The 39,584-participant group analysis also observed this inverse association. The results indicated no specific association between headache and type 2 DM.

The observed adverse association may reveal a protecting action of one status on the other, but the cross-sectional study cannot ascertain the direction of causality. Arteriosclerosis due to type 1 DM, considering vascular reactivity in the pathophysiology of migraine, and diabetic neuropathy caused by type 1 DM micro-vascular alteration (302) can be included as some explanations for minor migraine and headache prevalence in these patients. Insulin metabolism involved in migraine pathogenesis concluded in other studies (303), and some possible genetic factors (304, 305) may better not be neglected.

### Migraine and psoriasis

A meta-analysis by Rui Xu et al. in 2020 showed that migraine occurred severely in patients with psoriasis [pooled OR 1.64; 95% confidence interval (1.28; 2.11)]. This study indicated a significant association between migraine and psoriasis OR 1.64 (95% CI 1.28; 2.11). Although the underlying causes for this association have not been observed, pathophysiological, molecular, and therapeutic aspects should be considered explanatory factors (306). Migraine with aura (MA) is an independent risk factor for the cardiovascular system (CV) in people under 45 years of age (307, 308), and the number of MA crises is a sign of the severity of CV (309, 310). Patients with CV impairment also have high mortality due to psoriasis, indicating that MA can be a risk factor in psoriasis for CV. Anti-CGRP monoclonal antibodies are an innovative treatment for migraine relief (311). Biologic drugs that inhibit the TNF-α inactivate the pain-related signaling pathway in psoriatic arthritis (259), indicating a potential anti-inflammatory therapy target in migraine headaches. Evidence suggests that sterile inflammation in the intracranial meninges activates the trigeminal meningeal nociceptors (312). Due to this, a significant correlation and overlap of pro-inflammatory mediators in the neuromuscular and neuro-inflammatory mechanisms play an important role in migraine and psoriasis (312–314).

**Figure 4:**
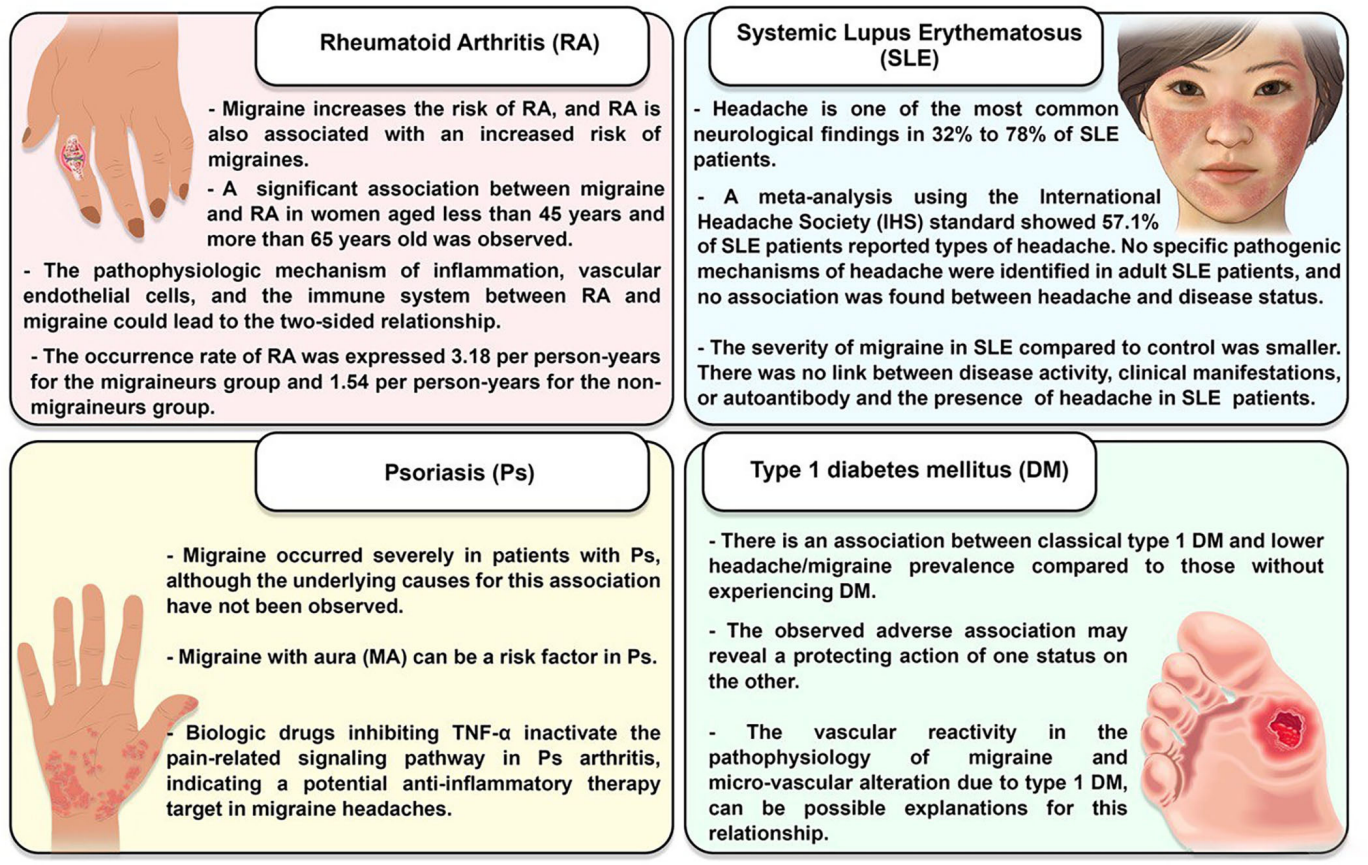
Migraine is a possible risk factor for RA, SLE, and proteus syndrome (Ps) but not T1DM.

Figure 4 summarizes the associations between migraine and RA, SLE, type 1 DM, and psoriasis.

## Conclusion

Evidence is provided in different literature indicating the immune system's involvement in migraine pathophysiology. A vital mechanism is a neuroinflammation which follows trigeminovascular afferents activation and sensitization. The afferents, which project to the brainstem second-order neurons, cause neuroinflammation and dilation of the meningeal vessels by locally releasing neurotransmitters and neuropeptides. Considering cytokines as potential mediators of pain in neurovascular inflammation, they may cause migraine pain generation. Cytokines can induce sterile inflammation of blood vessels in meninges in migraine. As mentioned, observations indicate a critical role of mast cells in migraine pathogenesis. Migraine triggers could directly or indirectly cause meningeal mast cells to activate the trigeminovascular system by releasing inflammatory mediators. CGRP involvement is essential in peripheral sensitization, neurogenic vasodilation, and migraine cascade initiation. The manifestation of headaches, especially migraines, is identified in many autoimmune disorders such as MS and SLE. Among other systemic autoimmune disorders, RA is more common in migraineurs than in people without migraine. The immunological system dysfunction could be a common pathophysiological relation between immunological disorders and migraine since it is suggested that some immunological dysfunction could involve the pathogenesis of migraine. Further trials investigating the effects of anti-inflammatory drugs on features of inflammation and pain in the context of migraine can provide a more profound view of more effective management of the disorder. Treatment of migraine would be better if it involved multidisciplinary approaches.

## Author contributions

Study concept, design, and critical revision of the manuscript for important intellectual content: ND and SY. Acquisition of data: MS, SP, DN, ZR, OS, and MR. Drafting of the manuscript: MS, SP, DN, ZR, OS, MR, SZ, MA, MT, AV, HS, MM, and SG. Study supervision: ND. All authors contributed to the article and approved the submitted version.

## Conflict of interest

The authors declare that the research was conducted in the absence of any commercial or financial relationships that could be construed as a potential conflict of interest.

## Publisher's note

All claims expressed in this article are solely those of the authors and do not necessarily represent those of their affiliated organizations, or those of the publisher, the editors and the reviewers. Any product that may be evaluated in this article, or claim that may be made by its manufacturer, is not guaranteed or endorsed by the publisher.
